# Synchronized Regulation of Different Zwitterionic Metabolites in the Osmoadaption of Phytoplankton

**DOI:** 10.3390/md11062168

**Published:** 2013-06-17

**Authors:** Björn Gebser, Georg Pohnert

**Affiliations:** Institute of Inorganic and Analytical Chemistry, Friedrich Schiller University, Lessingstr. 8, D-07743 Jena, Germany; E-Mail: Bjoern.Gebser@uni-jena.de

**Keywords:** *Emiliania huxleyi*, *Prorocentrum minimum*, dinoflagellates, haptophytes dimethylsulfoniopropionate (DMSP), gonyol, dimethylsulfide (DMS), osmoadaption

## Abstract

The ability to adapt to different seawater salinities is essential for cosmopolitan marine phytoplankton living in very diverse habitats. In this study, we examined the role of small zwitterionic metabolites in the osmoadaption of two common microalgae species *Emiliania huxleyi* and *Prorocentrum minimum*. By cultivation of the algae under salinities between 16‰ and 38‰ and subsequent analysis of dimethylsulfoniopropionate (DMSP), glycine betaine (GBT), gonyol, homarine, trigonelline, dimethylsulfonioacetate, trimethylammonium propionate, and trimethylammonium butyrate using HPLC-MS, we could reveal two fundamentally different osmoadaption mechanisms. While *E. huxleyi* responded with cell size reduction and a nearly constant ratio between the major metabolites DMSP, GBT and homarine to increasing salinity, osmolyte composition of *P. minimum* changed dramatically. In this alga DMSP concentration remained nearly constant at 18.6 mM between 20‰ and 32‰ but the amount of GBT and dimethylsulfonioacetate increased from 4% to 30% of total investigated osmolytes. Direct quantification of zwitterionic metabolites via LC-MS is a powerful tool to unravel the complex osmoadaption and regulation mechanisms of marine phytoplankton.

## 1. Introduction

Marine algae harbor different highly polar organic substances to maintain osmotic pressure balance with the surrounding sea water [1]. Organic osmolytes include polyols [2,3], amino acids [4,5] and zwitterionic substances [6,7,8]. Certain zwitterionic metabolites may fulfill multiple physiological roles in algae e.g., as osmolytes, antioxidants and cryoprotectants [9,10,11]. The zwitterionic dimethylsulfoniopropionate (DMSP) is considered to be a key player in this context and since it also serves as precursor for the volatile dimethylsulfide (DMS), it has attracted much attention [12,13,14]. DMS is among the most important sources of biogenic sulphur to the atmosphere contributing 13–37 Tg S emissions per year [15]. Another dominant zwitterionic osmolyte produced by marine phytoplankton is the nitrogen containing glycine betaine (GBT) [16]. In addition to these well studied osmolytes there are many other zwitterionic substances found in marine phytoplankton like gonyol [17], homarine [6], trigonelline [16], dimethylsulfonioacetate (DMS-Ac, dimethylthetin), trimethylammonium propionate (TMAP), and trimethylammonium butyrate (TMAB) with poorly understood physiological functions [6,18,19]. The interplay of all these potential osmoregulators under different salinities has hitherto not been addressed, which might be explained with methodological problems in the quantification of such small and highly polar metabolites. Recently we established a direct LC-MS method for simultaneous quantification of DMSP and glycine betaine, which is extended here for the comprehensive monitoring of all above mentioned zwitterionic metabolites [20,21].

**Scheme 1:**
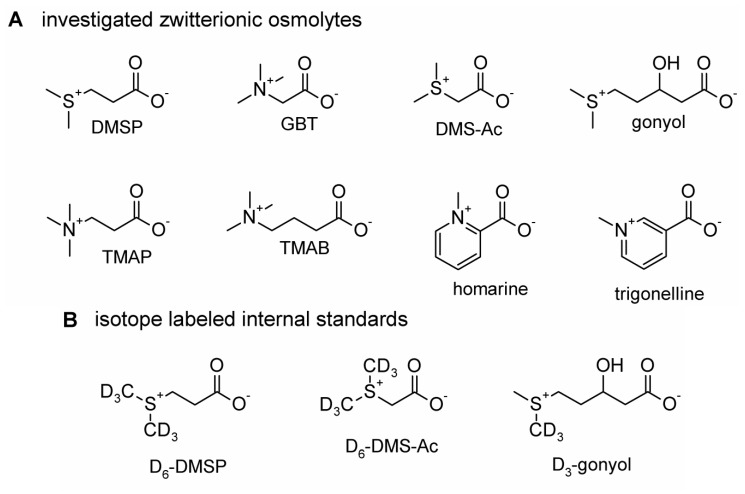
Chemical structures of zwitterionic osmolytes. (**A**) Investigated zwitterionic osmolytes: dimethylsulfoniopropionate (DMSP), glycine betaine (GBT), dimethylsulfonioacetate (DMS-Ac), gonyol, trimethylammonium propionate (TMAP), trimethylammonium butyrate (TMAB), homarine and trigonelline. (**B**) Isotope labeled internal standards: D_6_-dimethylsulfoniopropionate (D_6_-DMSP), D_6_-dimethylsulfonioacetate (D_6_-DMS-Ac) and D_3_-gonyol.

We investigated the composition of zwitterionic metabolites in two cosmopolitan algae after adaptation to different salinities. Due to their occurrence in diverse regions of the ocean these algae need efficient strategies to adapt to the variable seawater salinities in different habitats. We selected dominant algae that have become model organisms for the study of phytoplankton. The coccolithophore *Emiliania huxleyi* is present in all oceans except Arctic and Antarctic regions [22]. Also the dinoflagellate *Prorocentrum minimum* can be found in many regions like the North Atlantic [23,24], in Eastern Pacific [25] and the Baltic Sea [26]. Such a broad distribution in marine waters of different salinity requires efficient adaptation. In the present study we perform a comprehensive, simultaneous analysis of the zwitterionic metabolites DMSP, GBT, dimethylsulfonioacetate, gonyol, homarine, trigonelline, trimethylammonium propionate (TMAP) and trimethylammonium butyrate (TMAB) (Scheme 1). Adaptation to different salinities is achieved by a concerted adjustment of several different zwitterionic metabolites. In the two investigated species, osmoadaptation is reached by fundamentally different regulative processes. The osmolyte composition of phytoplankton is thus, not only species specific, but also variable under different conditions.

## 2. Results and Discussion

**Figure 1:**
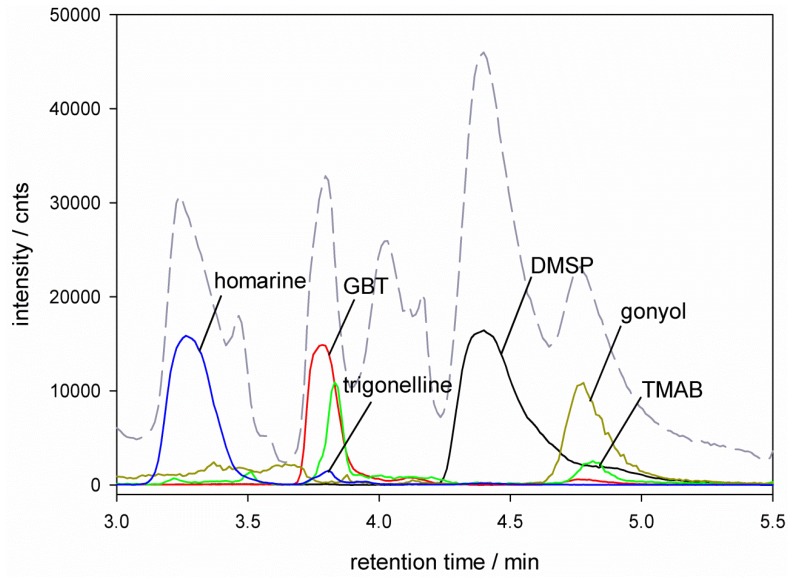
HPLC-MS separation of zwitterionic metabolites from *E. huxleyi* RCC1216. Total Ion Count (TIC --) and ion traces of dimethylsulfoniopropionate (DMSP), [M + 1] *m/z* = 135 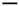), glycine betaine (GBT, [M + 1] *m/z* = 118 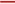), trimethylammonium butyrate (TMAB, [M + 1] *m/z* = 146 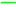), gonyol ([M + 1] *m/z* = 179 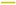), homarine and trigonelline ([M + 1] *m/z* = 138 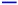). Ion traces of GBT, gonyol, TMAB and trigonelline are 10-times amplified.

**Figure 2:**
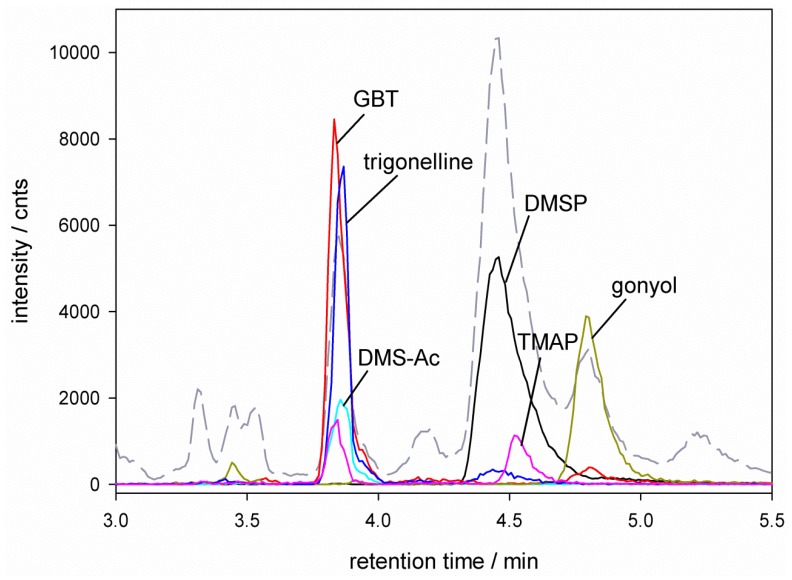
HPLC-MS separation of zwitterionic metabolites from *P. minimum*. Total Ion Count (TIC --) and ion traces of dimethylsulfoniopropionate (DMSP) ([M + 1] *m/z* = 135 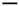), glycine betaine (GBT) ([M + 1] *m/z* = 118 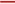), dimethylsulfonioacetate (DMS-Ac, [M + 1] *m/z* = 121 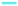), gonyol ([M + 1] *m/z* = 179 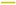), trigonelline ([M + 1] *m/z* = 138 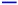) and trimethylammonium propionate (TMAP, [M + 1] *m/z* = 132 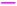). Ion traces of GBT, DMS-Ac, gonyol, TMAP, and trigonelline are 10-times amplified.

Several studies showed that the concentration of intracellular osmolytes in different algae species are highly dependent on their growth phase [16,27]. In order to avoid overlaying effects of adaptation to different salinities and growth phase, we ensured that every replicate sampling was performed at the beginning of the stationary phase. At this point all replicates are comparable and observed variations of the intracellular osmolyte concentrations can be attributed to the adaption to different salinities. Using established filtration and extraction protocols we could reliably generate samples for profiling of zwitterionic osmolytes [20,21]. Our previously introduced method of HPLC separation on a ZIC‑HILIC column and mass spectrometric detection using an ESI q-Tof-mass spectrometer allowed direct monitoring of DMSP and glycine betaine [20,21]. Since this method is highly sensitive for the detection of zwitterionic metabolites we can now extend its scope for the additional simultaneous monitoring of gonyol, DMS-acetate, homarine, trigonelline, trimethylammonium propionate (TMAP) and trimethylammonium butyrate (TMAB). HPLC did not allow a baseline separation of all mentioned analytes. The molecular ion traces of DMSP ([M + 1] *m/z* = 135), GBT ([M + 1] *m/z* = 118), DMS-Ac ([M + 1] *m/z* = 121), gonyol ([M + 1] *m/z* = 179), TMAP ([M + 1] *m/z* = 132), TMAB ([M + 1] *m/z* = 146), homarine ([M + 1] *m/z* = 138) and trigonelline ([M + 1] *m/z* = 138) could however be reliably integrated and quantified relative to the internal isotope labeled standard D_3_-gonyol. Figure 1, Figure 2 show example chromatograms and ion traces of *E. huxleyi* and *P. minimum* extracts grown in 26‰ salinity medium (ion traces of GBT, DMS-Ac, gonyol, TMAP, TMAB and trigonelline are 10-times amplified). The identity of all analytes was verified by co-injection with commercially available glycine betaine (Sigma Aldrich, Germany), trimethylammonium butyrate (Sigma Aldrich, Germany) and trigonelline (Sigma Aldrich, Germany). All other standard compounds used for quantification (DMSP, D_6_-DMSP, DMS-Ac, D_6_-DMS-Ac, gonyol, D_3_-gonyol, homarine and TMAP) were synthesized in our lab. The co-injection data and synthesis of standard compounds are documented in the supplementary material.

In order to allow adaptation of the algae to the respective salinities we inoculated a starting culture grown at 28‰ salinity for *P. minimum* (artificial seawater according to Maier and Calenberg [28]) and 30‰ salinity for *E. huxleyi* (HW sea salt medium according to Spielmeyer [29]) into three hyposaline (16‰, 20‰, 26‰) and two hypersaline (32‰ and 36‰ for *P. minimum* and 34‰ and 38‰ for *E. huxleyi*) media. Medium salinities between 30‰ and 34‰ mentioned above correspond to open ocean seawater conditions. Higher salinities of over 36‰ can be found in regions with high evaporation like the Red Sea or Mediterranean Sea. Hyposaline conditions correspond to regions with high freshwater influx like the Black Sea, Baltic Sea or estuarine areas of large rivers. Thus, chosen salinities in this study represent a selection of common conditions encountered in nature. The cultures were maintained with regular re-inoculation into the salinity adjusted media for four weeks. The fully adapted cultures were then used as starting stock for the experiments.

In order to monitor the growth of all replicates and to determine the beginning of the stationary phase for collecting samples, chlorophyll A fluorescence was measured at least every two days with daily measurements in later growth phases. Interestingly medium salinity had no obvious effect on the length of the exponential growth phase but affected maximum cell densities. All replicates reached the stationary phase within 17 days (±1 day) after inoculation (data shown in the supplementary material).

### 2.1. *Emiliania huxleyi*

In the survey of zwitterionic metabolites we detected the well-known osmolytes DMSP, gonyol and glycine betaine. In addition, we found homarine, trigonelline, TMAP and TMAB in lower amounts. The nitrogen containing zwitterionic homarine is described in several marine invertebrates [30,31] and in few microalgae like *Amphidinium carterae* and has previously also been detected in *E. huxleyi* (CCMP378) in low concentrations (0.2 mM to 0.5 mM) [16]. Trigonelline is not known as common metabolite from phytoplankton but has also been found in the microalgae *Chrysochromulina* sp., *Skeletonema costatum* and *Tetraselmis* sp. [16]. It was recently also detected in Laminariales brown algae [32,33]. TMAP and TMAB were to the best of our knowledge not described as natural products before. The sulfur containing DMS-acetate, known from other phytoplankton sources, was not detected.

**Figure 3:**
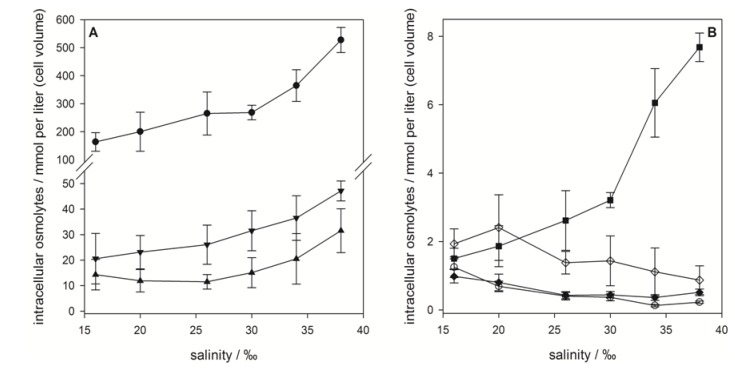
Intracellular concentrations of zwitterionic osmolytes of *E. huxleyi* RCC1216 as a function of medium salinity. (**A**) Major osmolytes: dimethylsulfoniopropionate (DMSP) (●), glycine betaine (GBT) (▲) and homarine (▼). (**B**) Minor osmolytes: gonyol (■), trimethylammonium propionate (TMAP) (♦), trimethylammonium butyrate (TMAB) (○) and trigonelline (◊). Concentrations are normalized to cell volume, error bars represent standard deviation (biological replicates, *N* = 5).

**Figure 4:**
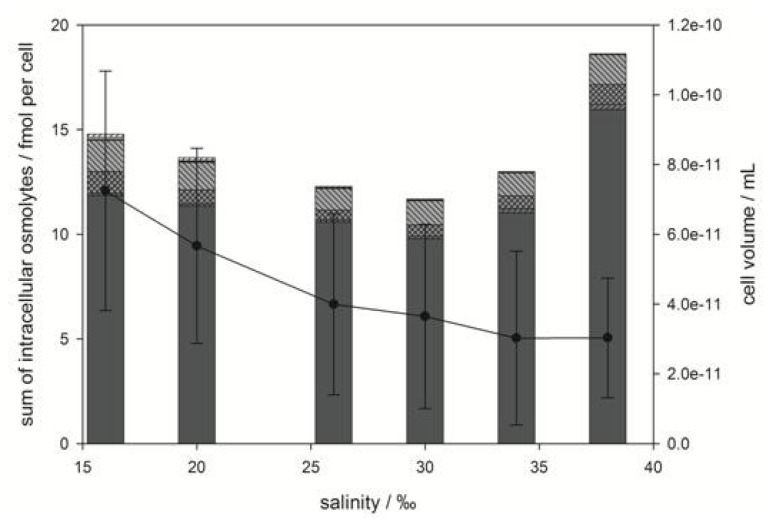
Total concentration of intracellular zwitterionic osmolytes (bar graph) and cell volume (line graph) of *E. huxleyi* as a function of medium salinity. Bars represent concentrations of dimethylsulfoniopropionate (DMSP) (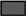), gonyol (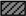), glycine betaine GBT (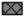), homarine (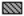), trimethylammonium propionate (TMAP) (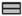), trimethylammonium butyrate (TMAB) (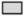) and trigonelline (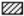). Osmolyte concentrations are normalized per cell, error bars represent standard deviation (biological replicates, *N* = 5).

Quantification of the sulfur containing osmolytes DMSP and gonyol and the nitrogen containing osmolytes GBT, homarine, TMAP, TMAB and trigonelline revealed that DMSP is the major zwitterionic osmolyte at all tested salinities. Intracellular concentrations ranged from 164 ± 33 mM (with respect to cell volume) at 16‰ medium salinity to 527 ± 44 mM at 38‰ (Figure 3A). The increase in concentration with rising salinity was more pronounced at higher values. The second most abundant zwitterionic osmolytes were GBT and homarine that were present in concentrations between 15.3 ± 6.0 mM at 16‰ and 31.5 ± 8.6 mM at 38‰ for GBT and 20.6 ± 9.9 mM at 16‰ and 47.2 ± 3.9 mM at 38‰ for homarine, respectively. The ratio between the major osmolytes, DMSP, GBT and homarine, remains at approximately 100:6:10 over the whole range of tested salinities suggesting that all pathways involved were regulated in similar ways. The less abundant gonyol also increased with increasing salinity (1.5 ± 0.3 mM at 16‰ to 7.7 ± 0.4 mM at 38‰), but a more pronounced increase was observed between the two highest salinities where the concentration at 38‰ was double that at 30‰ (Figure 3B). While the intracellular concentrations of DMSP, GBT, homarine and gonyol increased with increasing salinity in agreement with the expected behavior of osmolytes, the nitrogen containing compounds, TMAP, TMAB and trigonelline, all decreased in their concentration (Figure 3B). These minor abundant zwitterionic metabolites have not yet been described or quantified in algal cultures, but were possibly included as fractions of a complex mixture of quaternary ammonium compounds in previous surveys [7,8]. The decrease of intracellular TMAP, TMAB and trigonelline concentrations with increasing salinity, however, suggests that these zwitterionic metabolites play no role in osmoadaptation. Measurement of cell diameters and calculation of average cell volume revealed a considerable reduction of the cell volume with rising salinity (from 7.2 × 10^−11^ mL at 16‰ to 3.0 × 10^−11^ mL at 38‰) (Figure 4). In agreement with the literature this indicates that adjustment of the volume is an additional response to salinity changes [6,34]. If the concentration of zwitterionic compounds per cell is considered (Figure 4), it becomes clear that investment into the production of sulfur containing osmolytes is predominantly relevant at elevated salinity, while cell volume compensation seems to be more important at lower salinities.

Previous works regarding intracellular DMSP concentrations of *E. huxleyi* by Spielmeyer *et al.* (52 mM, HW sea salt medium, 30‰ salinity) [29], van Rijssel *et al.* (ca. 195 mM, artificial seawater according to Veldhuis) [35,36] and Keller (145 mM, K-medium, ca. 33‰ salinity, based on natural seawater) [16] show that intracellular DMSP concentrations can be highly dependent on the selected strain, cultivation conditions or sample processing procedures. Keller *et al.* also revealed differences in DMSP concentration between the growth stages of *E. huxleyi* (145 mM in exponential and 32.3 mM in stationary growth phase) [16]. Furthermore Bucciarelli *et al.* observed DMSP variation between 223 mM and 318 mM at the beginning and end of light period, respectively [37]. At an intermediate salinity of 30‰ a cellular DMSP concentration of 268 mM was found in this study confirming that our findings are within the range of literature data. Maximum DMSP concentrations of 527 mM measured in this study at the highest salinity of 38‰, which is about twice the concentration we found at a salinity of 30‰ illustrate the high influence of medium salinity on the concentration of this osmolyte. The large variations of detected concentrations between the different literature studies show that osmoadaption and regulation are very complex mechanisms and quantitative comparisons between different studies have to be treated with caution. It is therefore a particular strength of the current investigation that all osmolytes were recorded within a single experiment and one single extract, thereby minimizing inter-experiment variability and allowing the direct analysis of the response of multiple metabolites. GBT concentrations between 15.3 mM and 31.5 mM are in the same range as determined by Spielmeyer (20–35 mM) [21] and Keller (8.1–32.6 mM) [16]. In our study, homarine contributes significantly, with up to 10% to the total osmolyte content. While this metabolite was also detected in traces in *E. huxleyi* before an osmoregulatory function in this organism has not been described up to now [16]. Dickson and Kirst, however, showed a contribution to osmoregulation of homarine in the prasinophyte *Platymonas subordiformis* [6].

### 2.2. *Prorocentrum minimum*

**Figure 5:**
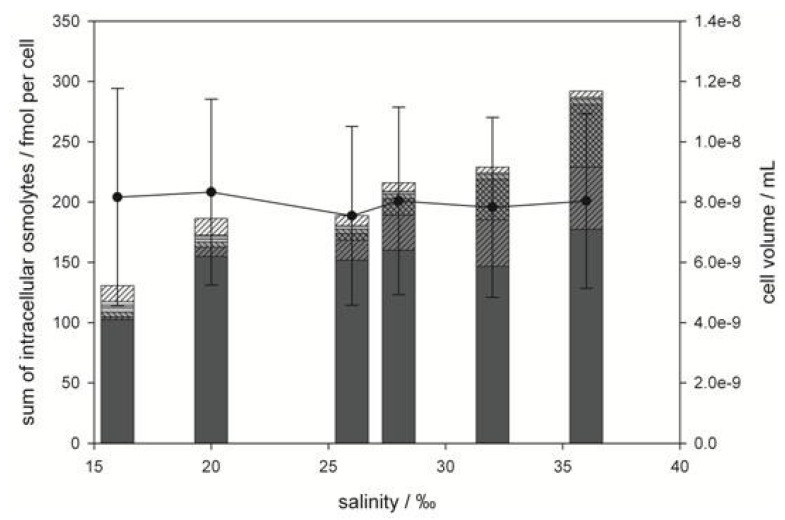
Total concentration of intracellular, zwitterionic osmolytes (bar graph) and cell volume (line graph) of *P. minimum* as a function of medium salinity. Bars represent concentrations of DMSP (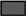), GBT (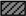), DMS-Ac (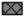), gonyol (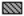), TMAP (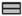), TMAB (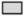) and trigonelline (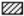). Osmolyte concentrations are normalized per cell, error bars represent standard deviation (biological replicates, *N* = 5).

In contrast to *E. huxleyi*, higher salt content of the medium had no effect on average cell size of *P. minimum* (Figure 5). Thus, adaptation to different salinities must rely on changes in osmolyte concentrations. However, the osmoadaptation of *P. minimum* has been poorly explored to date.

**Figure 6:**
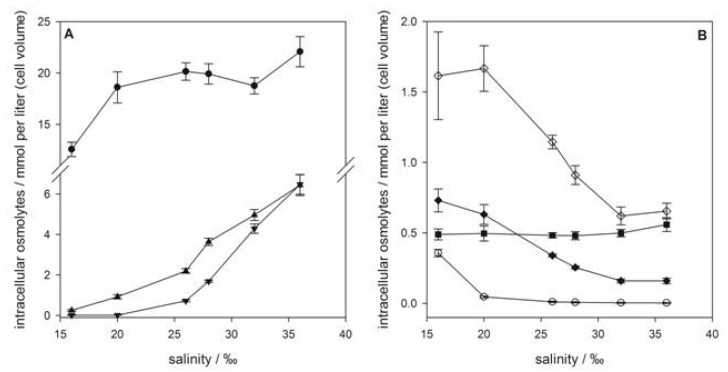
Intracellular concentrations of zwitterionic osmolytes of *P. minimum* as a function of medium salinity. (**A**) Major osmolytes: dimethylsulfoniopropionate (DMSP) (●), glycine betaine (GBT) (▲) and dimethylsulfonioacetate DMS-Ac (▼); (**B**) minor osmolytes: gonyol (■), trimethylammonium propionateTMAP (♦), trimethylammonium butyrateTMAB (○) and trigonelline (◊); concentrations are normalized to cell volume, error bars represent standard deviation (biological replicates, *N* = 5).

As in *E. huxleyi* DMSP was the dominant zwitterionic metabolite in *P. minimum* contributing 29.3%–40.0% of the investigated osmolytes at any given salinity. The composition of the other zwitterions was fundamentally different compared to *E. huxleyi*. At low salinities trigonelline was the second most abundant metabolite, while GBT and DMS-Ac increased with increasing salinity (Figure 6). The presence of GBT and trigonelline in *P. minimum* is in contrast to results by Keller *et al.* who did not detect these metabolites in this organism [16]. Gonyol, TMAP and TMAB were only detected in minute amounts (Figure 6B). The osmoadaptation of this dinoflagellate followed a fundamentally different pattern of zwitterion concentration changes compared to *E. huxleyi*. At the lowest salinity of 16‰, DMSP was by far the most abundant osmolyte with 12.6 ± 0.7 mM referred to cell volume (Figure 6A). Between 20‰ and 32‰ DMSP concentration remained almost constant at a level around 18.6 mM (concentration at 20‰). Only at the highest and lowest salinity tested was a change in concentration of DMSP observed (Figure 6A).

In contrast GBT and DMS-Ac concentration increased dramatically from 0.25 ± 0.04 mM to 4.96 ± 0.27 mM and from not detectable concentrations to 4.29 ± 0.23 mM, respectively. This suggests that DMSP is maintained at a high but constant level under average salinities and only plays a role in osmoadaption under extreme salinity conditions. The significant increase of GBT and DMS-Ac with increased salinity contributes most to the changes of the osmolyte composition of *P. minimum* (Figure 5). This suggests that GBT and DMS-Ac are highly regulated if adaptation to intermediate salinity changes is concerned. Regarding the minor abundant zwitterionic metabolites it could be shown that gonyol concentration remains constant at 0.49 ± 0.05 mM over the whole salinity range (Figure 6B). Concentrations of TMAP, and trigonelline decreased from 0.731 ± 0.081 mM at 16‰ to 0.159 ± 0.019 mM at 36‰ and from 1.62 ± 0.31 mM at 16‰ to 0.654 ± 0.055 mM at 36‰, respectively. Also, the concentration of TMAB dropped from 0.357 ± 0.026 mM at 16‰ to 0.046 ± 0.007 mM at 20‰, corresponding to a decrease of 87%. TMAP was not detectable at higher salinities. These metabolites did not, therefore, contribute significantly to the overall changes of the osmolyte pool (Figure 5).

The pronounced changes in osmolyte composition of *P. minimum* are in accordance with a lack of adjustment of cell size under different salinities. Compensation thus solely depends on osmolytes, which are orchestrated in an unexpected way. Apparently, fundamentally different metabolic pathways are involved in the adaptation of the two investigated model algae; how these are addressed remains open and will be subject to further studies. Besides the investigated osmolytes, numerous other metabolites can be affected by salinity changes. Some metabolites might have obvious function in osmoregulation, while others, such as the toxins of different harmful algae like cyanobacterium *Anabaena* sp. [38] and the dinoflagellate *Protoceratium reticulatum* [39], are also affected by salinity changes. These compounds might be rather affected via indirect influences of other metabolic pathways.

## 3. Experimental Section

### 3.1. Cultivation of Microalgae

Medium for cultures of *Emiliania huxleyi* (obtained from the Roscoff Culture Collection, Roscoff, France, strain number RCC1216) was prepared according to Spielmeyer *et al.* [20]. Concentration of the sea salt (HW sea salt professional, aquaristik.net, Babenhausen, Germany) was modified to obtain salinity of 16‰, 20‰, 26‰, 30‰, 34‰ and 38‰, respectively. The amount of added nutrients was not changed.

*Prorocentrum minimum* was cultivated in artificial seawater medium according to Maier and Calenberg [28]. Salinity of the medium was adjusted by modifying the content of the main salts mix, resulting in salinities of 16‰, 20‰, 26‰, 28‰, 32‰ and 36‰. At 28‰ salinity the main salts were NaCl (24 g/L), MgSO_4_∙7H_2_O (8 g/L), KCl (0.75 g/L), CaCl_2_·2H_2_O (1.5 g/L), HEPES (1.2 g/L), NaHCO_3_ (0.2 g/L). Concentration of trace metals and vitamins were not changed. Final salinities of the media were measured using a portable refractometer with automatic temperature compensation (VWR, Germany). All media were autoclaved before use.

Microalgae were cultivated as standing cultures in 50 mL polystyrene cell culture bottles (Carl Roth GmbH, Germany) with membrane filter screw caps for gas exchange. Stock cultures of *E. huxleyi* and *P. minimum* were adapted to the different salinities by cultivation in the respective media over a period of four weeks. During this time, cultures were diluted 1:1 every fourth day to maintain an exponential growth phase. After adaption to the different salinities, these cultures were used for inoculation of all biological replicates used for osmolyte quantification. Initial cell densities for the experiment were 4 kcells/mL for *P. minimum* and 20 kcells/mL for *E. huxleyi* respectively. For each salinity, five biological replicates were prepared. All cultures were grown at a temperature of 14 °C ± 2 °C with a 14:10 light:dark cycle. Light was provided by Osram biolux lamps with an intensity of 40 μmol photons m^−2^s^−1^.

The growth stages of every replicate were determined by daily measurement of *in vivo* fluorescence of a 200 μL sample using a microplate reader (Mithras LB 940, Berthold Technologies, Bad Wildbad, Germany). The excitation and emission wavelengths were set to 430 nm and 655 nm, respectively.

### 3.2. Cell Counting and Size Measurement

For determination of the final cell densities, *E. huxleyi* samples were counted after fixation with glutaraldehyde using a Cytomics FC 500 flow cytometer (Beckman Coulter, Krefeld, Germany) with CXP-software, air-cooled argon-ion laser (20 mW, 488 nm) and standard filters. The discriminator was set to side scatter and samples were analyzed for 1 min at a flow rate of 30 μL/min. Data were normalized to 3.6 μm polystyrene beads (Beckman Coulter, Krefeld, Germany) measured at 620 nm using CXP analysis software. *P. minimum* samples were fixed with Lugol and counted in Fuchs-Rosenthal chamber using a Leica DM2000 (Heerbrugg, Switzerland) upright microscope with phase contrast.

Pictures for cell size measurements were taken with a Leica DFC280 system. Average cell diameters were calculated using 200 randomly taken pictures of one replicate for every salinity to obtain representative data. Calculations of the average cell volumes based on a spherical shape, including the coccosphere for *E. huxleyi* and an oblate spheroid for *P. minimum*, whose minor axis was calculated from average diameter *vs.* height ratios of 50 different cells.

### 3.3. Sample Preparation

Samples for measurement were taken at the beginning of the stationary phase (6 h after beginning of the light period) by vacuum filtration of 25 mL of the cell culture on Whatman GF/C filters (700 mbar). The filters were immediately transferred into 15 mL Falcon^®^ tubes, and 2 mL of methanol for extraction of osmolytes was added. After 30 min at room temperature, extracts were stored at −80 °C. After thawing, samples were centrifuged for 2 min at 6.000 rcf and 100 μL of the supernatant were diluted with 900 μL acetonitrile and 100 μL of an aqueous solution of an internal standard mixture (D_3_-gonyol, D_6_-DMSP, D_6_-DMSAc). After centrifugation (5 min, 16000 rcf), the supernatant was directly used for ultra performance liquid chromatography (UPLC) analysis. 

### 3.4. Equipment

For analytical separation an Aquitiy UPLC (Waters, Milford, MA, USA) equipped with a SeQuant ZIC^®^-HILIC column (5 μm, 2.1 × 150 mm, SeQuant, Umeå, Sweden) and a SeQuant ZIC^®^-HILIC guard column (5 μm, 2.1 × 20 mm, SeQuant, Umeå, Sweden) was used. A Q-ToF micro mass spectrometer (Waters Micromass, Manchester, England) with electrospray ionization was used as a mass analyzer.

### 3.5. Osmolyte Analysis

For separation of osmolytes via UPLC we used the method of Spielmeyer [20] with water + 2% acetonitrile and 0.1% formic acid (solvent A) and 90% acetonitrile + 10% water with 5 mmol/L ammonium acetate (solvent B) as eluent system. The flow rate was set to 0.60 mL/min. As internal standards for quantification of DMSP and DMS-Ac we used D_6_-DMSP and D_6_-DMS-Ac, respectively. For all other osmolytes, we used D_3_-gonyol as internal standard. For proper quantification of all examined zwitterionic substances, relative response factors were determined by measurement of an equimolar mixture of all used labeled and unlabeled compounds. Response factors were calculated by comparison of the peak area of the analytes with the peak area of the corresponding internal standard.

Syntheses of standard compounds that are not commercially available are described in the supplementary materials.

## 4. Conclusions

LC-MS analysis of zwitterionic metabolites provides a powerful tool to unravel osmoregulation and osmoadaption mechanisms for a better understanding of the ability and success of cosmopolitan living algae to deal with a wide spectrum of salinities in different habitats. With simultaneous analysis of a large set of zwitterionic metabolites in the coccolithophore *E. huxleyi* and the dinoflagellate *P. minimum,* we were able to distinguish between two completely different strategies of osmoadaption of these two marine algae. While *E. huxleyi* reacts with a lower cell size to higher salinities to overcome osmotic stress, cell volumes of *P. minimum* remained constant over the whole range of salinity (16‰–38‰). The ratio of the main osmolytes in *E. huxleyi* remained more or less constant, which underlines the strategy of osmoadaption by cell size adjustment, whereas osmolyte composition and cellular concentrations in *P. minimum* changed in a pronounced way with increasing salinity. Therefore, intracellular concentrations of zwitterionic osmolytes and the whole osmolyte composition are not just species specific, but also highly dependent on the salinity of the culture medium and consequently, on the marine habitat.

## Supplementary Files

Supplementary File 1 Supplementary Materials (PDF, 569 KB)
