# Supplementary material for: Synchronized Regulation of Different Zwitterionic Metabolites in the Osmoadaption of Phytoplankton
Source: Mar Drugs. 2013 Jun 17;11(6):2168–82. doi: 10.3390/md11062168 (PMC3721227; doi:10.3390/md11062168)
Supplement: Supplementary File 1 — Supplementary Materials (PDF, 569 KB) [file marinedrugs-11-02168-s001.pdf]

Supplementary Materials

S1. Synthesis of Standard Compounds

S1.1. DMSP and D_6-DMSP

DMSP and D_6-DMSP as hydrochloride were synthesized according to Chambers [1] by bubbling gaseous hydrogen chloride through a solution of anhydrous acrylic acid (Fluka, Germany) and dimethyl sulfide (Sigma Aldrich, Germany) or D_6-dimethyl sulfide (Sigma Aldrich, Germany) in dichloromethane and subsequent recrystallization of the resulting white solid in MeOH/Et_2O.

S1.2. DMS-Ac and D_6-DMS-Ac

DMS-Ac and D_6-DMS-Ac as hydrobromide were synthesized according to Howard [2] by addition of commercially available dimethyl sulfide (Sigma Aldrich, Germany) and D_6-dimethyl sulfide (Sigma Aldrich, Germany), respectively, to a stirred solution of bromoacetic acid (Fluka, Germany) in dichloromethane. The resulting white solid was recrystallized in MeOH/Et_2O.

S1.3. Trimethylammonium Propionate

To a stirred solution of 100 mg of dimethylaminopropionic acid in 2 mL methanol were added 50 mg K_2CO_3 and 200 mg iodomethane (Sigma Aldrich, Germany) at room temperature. After two days, the resulting mixture was acidified by addition of aqueous hydroiodic acid (Alfa Aesar, Germany). Trimethylammonium propionate as hydroiodide was precipitated by addition of Et_2O as white solid, which was recrystallized in MeOH/Et_2O.

^{1}H-NMR (400 MHz, D_2O) δ ppm: 2.94 (2H, t, *J* = 7.68 Hz), 3.12 (9H, s), 3.64 (2H, t, *J* = 7.50 Hz); ^{13}C-NMR (50 MHz, D_2O) δ ppm: 27.75, 52.75, 61.27, 172.98; ESI-MS *m/z* 132.12 $[M + H]^+$; ESI-MS-MS (parent ion: *m/z* 132, collision energy: 15 eV) *m/z* 132.12 $[M]^+$, 73.08 $[M - C_3H_9N]^+$, 60.13 $[C_3H_{10}N]^+$, 59.12, 58.11.

*S1.4. Homarine (*N*-Methyl Picolinic Acid Hydroiodide)*

N-methylpicolinic acid hydroiodide was synthezised by addition of 150 mg iodomethane (Sigma Aldrich, Germany) to a well stirred suspension of 100 mg picolinic acid (Alfa Aesar, Germany) in 2 mL propylene carbonate. After two days, 10 mL Et_2O were added, and the resulting yellow solid was recrystallized in MeOH/Et_2O (for synthesis see also [3]).

^{1}H-NMR (400 MHz, MeOD) δ ppm: 4.95 (3H, s), 8.06 (1H, t, *J* = 6.26 Hz), 8.44 (1H, d, *J* = 7.63 Hz), 8.51 (1H, t, *J* = 7.78 Hz), 8.85 (1H, d, *J* = 5.19 Hz); ^{13}C-NMR (101 MHz, MeOD) δ ppm: 127.97, 130.51, 145.44, 146.71, 149.69, 163.90 (the signal of the methyl group was overlapped by the signals of the solvent and therefore not visible, cf. [4]); ESI-MS (positive) *m/z* 138.08$[M + H]^+$; ESI-MS-MS (positive, parent ion: *m/z* 138, collision energy: 15 eV) *m/z* 138.08 $[M + H]^+$, 124.09 $[M - CH_3 + H]^+$, 106.08, 96.09, 94.11 $[M - COOH + H]^+$, 78.08.

S1.5. Ethyl-3-hydroxy-5-methylthiopentanoate

In a three-necked flask (100 mL) with 5 g zinc powder 40 mL dry diethylether and 0.3 mL chlorotrimethylsilane were added under argon atmosphere. The resulting suspension was refluxed for 20 min. After dropwise addition of 3.3 g (19.8 mmol) ethyl-bromoacetate and 2.02 g (19.4 mmol) 3-(methylthio)propionaldehyde reaction mixture was refluxed over an additional 2.5 h. After cooling to room temperature 50 mL 3 M HCl were added and the resulting mixture was stirred for 20 min. The solution was then extracted with Et_2O and combined organic phases were washed with $NaHCO_3$ solution and water. Removal of the solvent gave a yellowish residue which was purified by silica gel column chromatography (petroleum ether/AcOEt = 4:1) to give 937 mg (25%) of the desired product as a colorless oil.

^{1}H-NMR (400 MHz, $CDCl_3$) δ ppm: 1.26 (3H, t, *J* = 7.14 Hz), 1.64–1.87 (2H, m), 2.10 (3H, s), 2.36–2.54 (2H, m), 2.55–2.71 (2H, m), 4.06–4.22 (3H, m); ^{13}C-NMR (50 MHz, $CDCl_3$) δ ppm: 14.10, 15.46, 30.33, 35.53, 41.22, 60.69, 66.88, 172.62; EIMS *m/z* (relative intensity, 70 eV) 192.08 $[M]^+$ (18), 174.07 $[M - H_2O]^+$ (23) , 144.08 (29), 129.03 $[M - H_2O - C_2H_5O]^+$ (46), 107.07 (32), 100.04 (22), 99.04 (16), 98.03 (39), 87.02 (21), 85.02 (20), 75.03 $[M - C_5H_9O_3]^+$ (20), 71.02 (28), 70.04 (22), 61.01 $[M - C_6H_{11}O_3]^+$ (100).

S1.6. 3-Hydroxy-5-methylthiopentanoic Acid

To 475 mg Ethyl-3-hydroxy-5-methylthiopentanoate in a 4 mL screw cap vial, 2 mL 10% NaOH solution was added and shaken for 30 min at room temperature until the mixture became a homogeneous solution. After washing with Et_2O the yellowish solution was acidified with conc. HCl to pH 1. The aqueous phase was extracted five times with 2 mL Et_2O. Removal of the solvent gave 275 mg (63%) of an orange, viscous liquid.

^{1}H-NMR (400 MHz, $CDCl_3$) δ ppm: 1.68–1.91 (2H, m), 2.11 (3H, s), 2.47–2.58 (2H, m), 2.58–2.67 (2H, m), 4.15–4.25 (1H, m), 5.90 (1H, br. s); ^{13}C-NMR (50 MHz, $CDCl_3$) δ ppm: 15.44, 30.32, 35.29, 41.07, 66.99, 176.91; ESI-MS (negative) *m/z* 163.09 $[M - H]^-$.

S1.7. D_3-Gonyol (as Hydroiodide)

100 mg of 3-hydroxy-5-methylthiopentanoic acid were dissolved in 1 mL acetone. After addition of 0.05 mL Iodomethane-d_3 the reaction mixture was stirred for 12 h at room temperature while an orange oily liquid precipitated out of the solution. After removal of the solvent under reduced pressure and reprecipitation of the resulting residue in MeOH/Et_2O 145 mg (77%) of D_3-gonyol hydroiodide were obtained.

^{1}H-NMR (400 MHz, MeOD) δ ppm: 1.89–2.01 (1H, m), 2.07–2.17 (1H, m), 2.47–2.60 (2H, m) 2.94, 2.95 (3H, ss), 3.37–3.53 (2H, m), 4.10–4.19 (1H, m); ^{13}C-NMR (101 MHz, MeOD) δ ppm: 25.84, 26.19, 31.98, 42.40, 42.77, 67.83, 174.78; ESI-MS (positive) *m/z* 182.02 $[M + H]^+$; ESI-MS-MS (positive, parent ion: *m/z* 182, collision energy: 15 eV) *m/z* 182.02 $[M + H]^+$, 117.11 $[M + H - C_2H_3D_3S]^+$, 99.10 $[M + H - H_2O - C_2H_3D_3S]^+$, 89.07, 87.10, 75.09, 71.10, 66.09 $[C_2H_4D_3S]^+$, 57.08, 55.10.

S2. Identification of Different Osmolytes

Chemical structures of all previously unknown osmolytes in cell extracts were verified by ESI-MS-MS experiments and co-injection with standard compounds. Collision energy for all ESI-MS-MS experiments was set to 15 eV. In the following ion traces of investigated osmolytes of cell extracts (solid lines), standard compounds (dashed lines) and corresponding mass spectra are shown.

S2.1. DMS-Acetate

Figure S1. Ion traces (*m/z* = 121) of dimethylsulphonioacetate (DMS-Ac) in cell extracts (—), corresponding standard compound (--) and ESI-MS-MS spectrum of DMS-Ac (parent ion: *m/z* = 121).

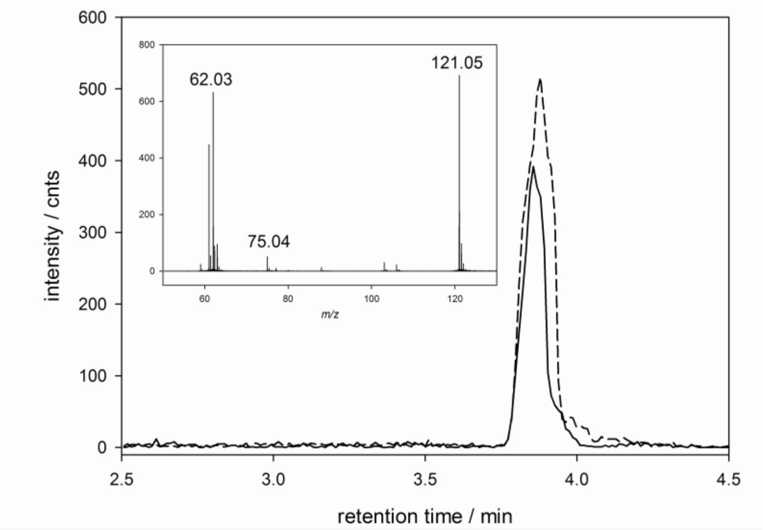

S2.2. TMAP

Figure S2. Ion traces (*m/z* = 132) of trimethylammoniumpropionate (TMAP) in cell extracts (—), corresponding standard compound (--) and ESI-MS-MS spectrum of TMAP (parent ion: *m/z* = 132).

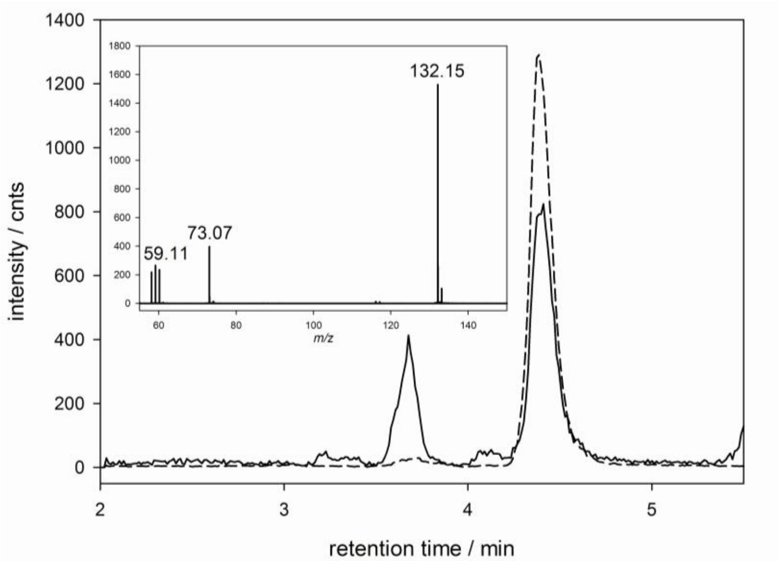

S2.3. TMAB

Figure S3. Ion traces (*m/z* = 146) of trimethylammoinimbutyrate (TMAB) in cell extracts (—), corresponding standard compound (--) and ESI-MS-MS spectrum of TMAB (parent ion: *m/z* = 146).

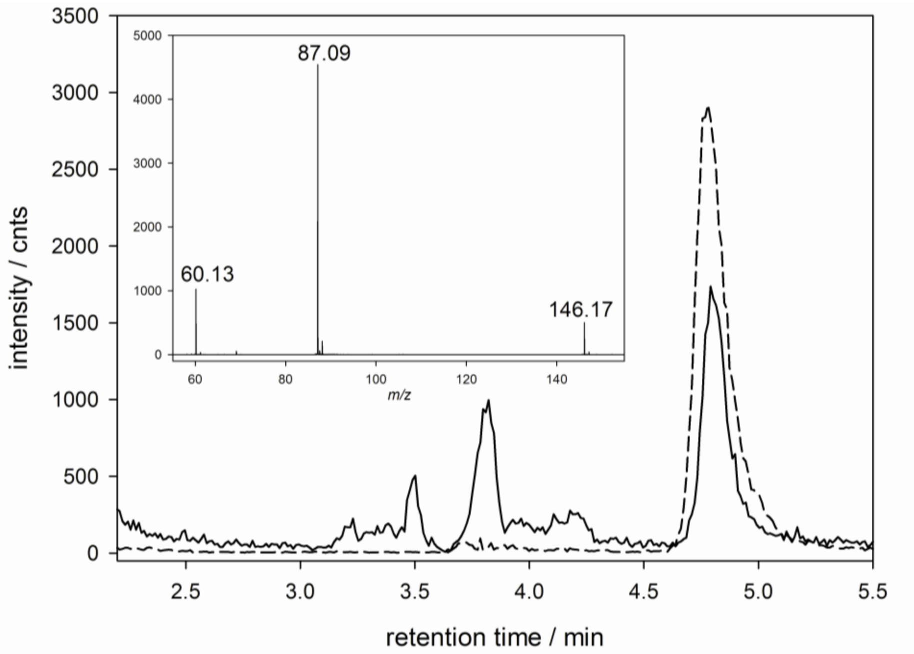

S2.4. Trigonelline

Figure S4. Ion traces (*m/z* = 138) of trigonelline in cell extracts (—), corresponding standard compound (--) and ESI-MS-MS spectrum of trigonelline (parent ion: *m/z* = 138).

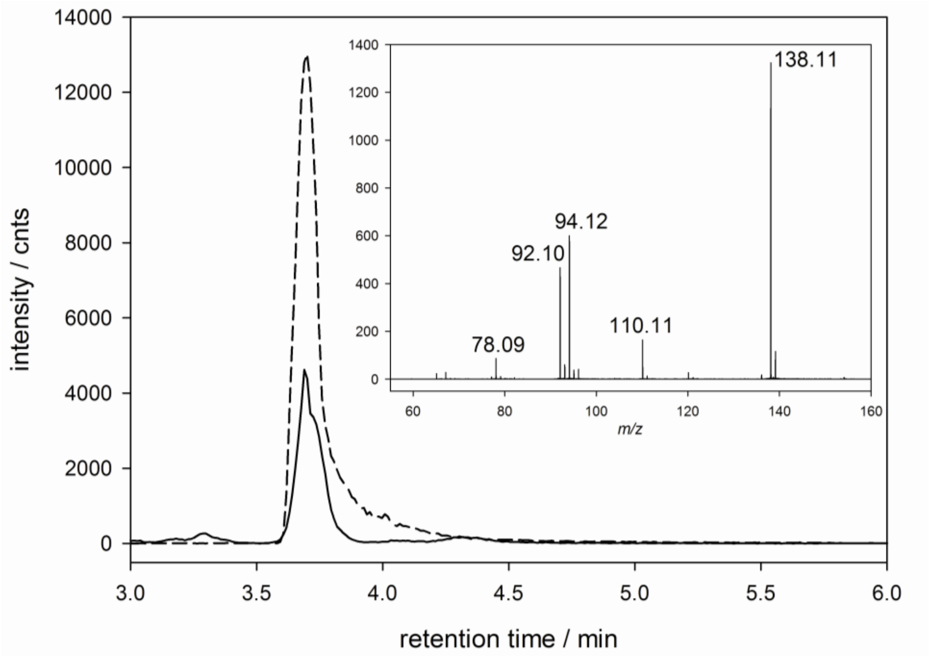

S2.5. Homarine

Figure S5. Ion traces (*m/z* = 138) of homarine in cell extracts (—), corresponding standard compound (--) and ESI-MS-MS spectrum of homarine (parent ion: *m/z* = 138).

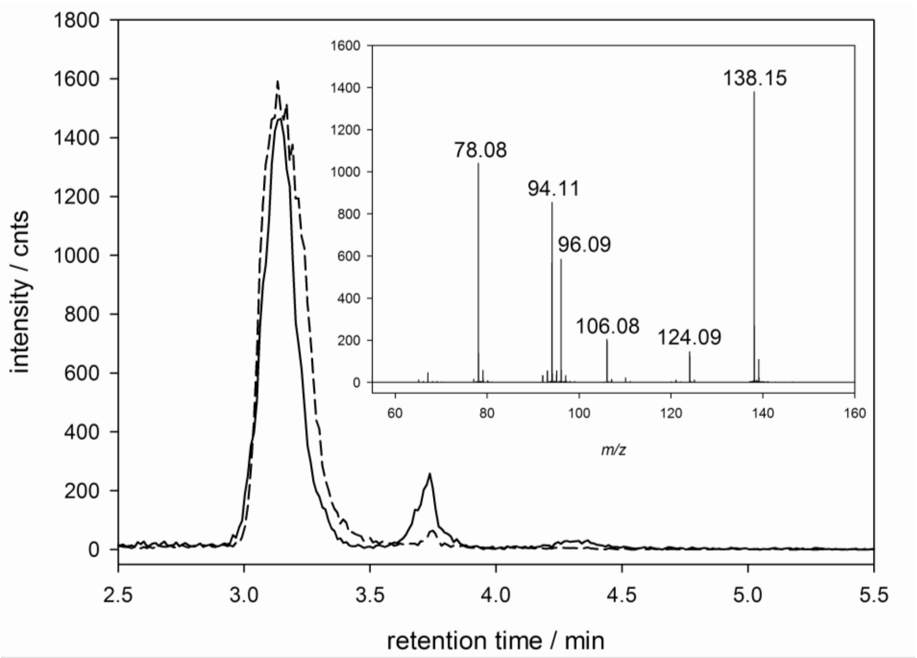

S3. Growth Curves (*in Vivo* Chlorophyll-A Fluorescence Data)

Figures S6 and S7 show measured *in vivo* chlorophyll-A fluorescence of *E. huxleyi* and *P. minimum* over the growth curve. Last data point represents the day of sample collection and extraction of the algae.

Figure S6. *In vivo* chlorophyll-A fluorescence during growth curve of *E. huxleyi* RCC1216 cultures grown in HW sea salt medium with salinities of 16‰ (—), 20‰ (—), 26‰ (—), 30‰ (—), 34‰ (—) and 38‰ (—); error bars represent standard deviation between biological replicates, last data point is day of sample collection and cell extraction.

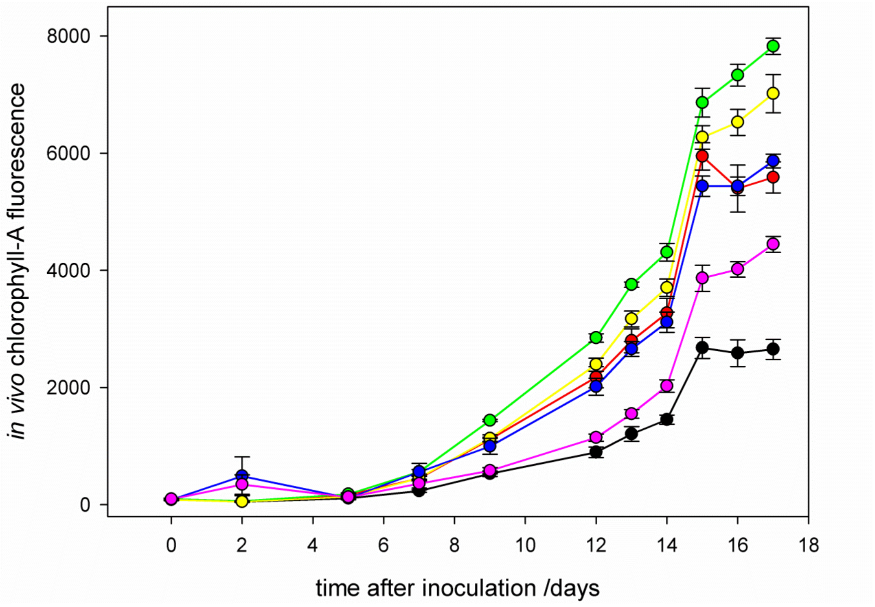

Figure S7. *In vivo* chlorophyll-A fluorescence during growth curve of *P.minimum* cultures grown in artificial seawater with salinities of 16‰ (—), 20‰ (—), 26‰ (—), 28‰ (—), 32‰ (—) and 36‰ (—); error bars represent standard deviation between biological replicates, last data point is day of sample collection and cell extraction.

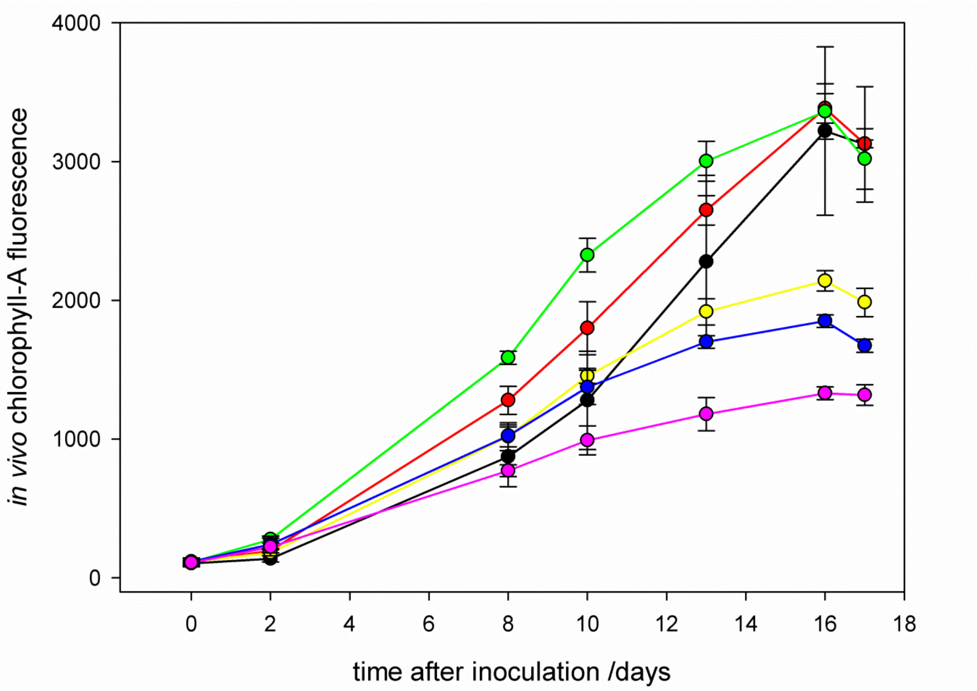

References

1. Chambers, S.T.; Kunin, C.M.; Miller, D.; Hamada, A. Dimethylthetin can substitute for glycine betaine as an osmoprotectant molecule for *Escherichia coli*. *J. Bacteriol.* **1987**, *169*, 4845–4847.
2. Howard, A.G.; Russell, D.W. Borohydride-coupled HPLC-FPD instrumentation and its use in the determination of dimethylsulfonium compounds. *Anal. Chem.* **1997**, *69*, 2882–2887.
3. McClintock, J.B.; Baker, B.J.; Hamann, M.T.; Yoshida, W.; Slattery, M.; Heine, J.N.; Bryan, P.J.; Jayatilake, G.S.; Moon, B.H. Homarine as a feeding deterrent in common shallow-water antarctic lamellarian gastropod *Marseniopsis mollis*: A rare example of chemical defense in a marine prosobranch. *J. Chem. Ecol.* **1994**, *20*, 2539–2549.
4. Pénez, N.; Culioli, G.; Pérez, T.; Briand, J.-F.; Thomas, O.P.; Blache, Y. Antifouling properties of simple indole and purine alkaloids from the mediterranean gorgonian *Paramuricea clavata*. *J. Nat. Prod.* **2011**, *74*, 2304–2308.

© 2013 by the authors; licensee MDPI, Basel, Switzerland. This article is an open access article distributed under the terms and conditions of the Creative Commons Attribution license (http://creativecommons.org/licenses/by/3.0/).
